# Tumor‐Bearing Status Accelerates Bleomycin‐Induced Pulmonary Inflammation via Endothelial Activation

**DOI:** 10.1111/1759-7714.70272

**Published:** 2026-03-30

**Authors:** Shoko Isoyama, Kakuhiro Yamaguchi, Hiroshi Iwamoto, Yasushi Horimasu, Kunihiko Funaishi, Kiyofumi Shimoji, Shinjiro Sakamoto, Takeshi Masuda, Taku Nakashima, Shinichiro Ohshimo, Hironobu Hamada, Noboru Hattori

**Affiliations:** ^1^ Department of Molecular and Internal Medicine, Graduate School of Biomedical and Health Science Hiroshima University Hiroshima Japan; ^2^ Department of Respiratory Medicine Hiroshima University Hospital Hiroshima Japan; ^3^ Department of Emergency and Critical Care Medicine, Graduate School of Biomedical and Health Science Hiroshima University Hiroshima Japan; ^4^ Department of Physical Analysis and Therapeutic Sciences, Graduate School of Biomedical and Health Science Hiroshima University Hiroshima Japan

**Keywords:** drug‐induced lung disease, endothelial cells, HMGB1, inflammation, tumor

## Abstract

**Background:**

Drug‐induced lung disease (DILD) is a severe adverse event of cancer treatment. Several clinical reports have demonstrated an association between DILD and tumor progression. However, the underlying mechanism remains unclear. This study aimed to elucidate the role of tumor‐bearing status in the development of DILD.

**Methods:**

We prepared a subcutaneous Lewis lung carcinoma (LLC) and KLN205‐bearing model. To trigger DILD, bleomycin (BLM) was administered subcutaneously. mRNA expression associated with endothelial activation (PAI‐1, vWF, and ICAM‐1), inflammatory cell infiltration, and alveolar wall thickness was assessed by using bronchioalveolar lavage fluid (BALF) and lung tissue. Additionally, the role of high‐mobility group box 1 (HMGB1) in tumor‐bearing status was examined.

**Results:**

Compared with control mice, LLC‐ and KLN205‐bearing mice showed a tendency toward increased expression of at least one of PAI‐1, vWF, and ICAM‐1 on endothelium, along with inflammatory cell infiltration in the lungs. BLM‐treated mice with LLC showed more inflammatory cell infiltration than BLM‐treated mice, accompanied by a significant increase in PAI‐1, vWF, and ICAM‐1 expression on endothelium. Moreover, BLM‐treated mice with LLC exhibited pronounced alveolar wall thickening. In LLC‐bearing mice, serum HMGB1 levels were significantly higher compared with control mice. Additionally, inflammatory cell infiltration in the lungs tended to be increased by the intraperitoneal injection of HMGB1, which was accompanied by increased expression of vWF and ICAM‐1 on endothelium.

**Conclusions:**

This study showed that tumor‐bearing status elicits proinflammatory activation in endothelial cells and inflammatory cell infiltration into the lungs that aggravates DILD caused by BLM.

## Graphical abstract

In subcutaneous tumor‐bearing mice, inflammatory cell infiltration into the lung was aggravated via endothelial activation. The tumor‐bearing status additively aggravated BLM‐induced lung inflammation. Proinflammatory changes in the lungs accelerated by tumor‐bearing status might be involved in the development of drug‐induced lung inflammation.

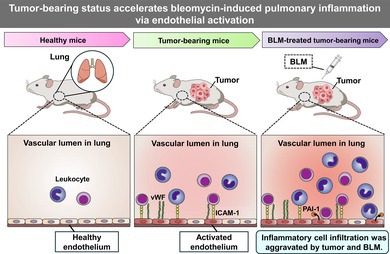

## 1 Introduction

Drug‐induced interstitial lung disease (DILD) is defined as a lung injury resulting from exposure to drugs that cause inflammation, and possibly interstitial fibrosis [1, 2]. Although the pathogenic mechanisms of DILD remain unclear, both cytotoxic and immune mechanisms may be involved independently or in conjunction during its initiation and propagation [1, 3]. Cytotoxic chemotherapy in cancer patients is one of the representative causes of DILD associated with cytotoxic mechanisms [3]. Chemotherapeutic agents can directly damage pneumocytes and the alveolar capillary endothelium, thereby promoting the release of cytokines and recruitment of inflammatory cells [3]. Although DILD is a potential complication following chemotherapy and can sometimes be fatal [4, 5, 6], cytotoxic drugs do not cause life‐threatening lung injury in all patients with cancer. Therefore, the mechanism of DILD needs to be elucidated by focusing not only on the inherent toxicity of these drugs but also on the specific conditions of individual patients, which aggravate the toxicity.

DILD is likely modified by a variety of host and environmental factors, including genetic predisposition, age, interactions with concomitant drugs, and underlying pathological conditions in the lungs, particularly pulmonary fibrosis, and chronic inflammatory lung disease [7]. Especially in patients with cancer, a history of cigarette smoking, preexisting lung disease, previous DILD, poor performance status, and advanced stage of underlying cancer have been reported as risk factors for the development of DILD [8]. Regarding the association between chemotherapy‐related DILD and advanced stages of underlying cancer, one representative report has shown that the presence of distant metastasis is associated with an increased incidence of cytotoxic chemotherapy‐induced lung injury [9]. In line with this report, we have reported that a higher tumor volume before treatment is associated with an earlier onset of cytotoxic chemotherapy‐induced lung injury in patients with lung cancer and preexisting interstitial lung disease [10]. However, the mechanistic association between cancer and the aggravation of lung injury remains unclear.

Furthermore, higher tumor burden is significantly associated with higher serum levels of high‐mobility group box 1 (HMGB1), and higher levels of serum HMGB1 before treatment are associated with a higher incidence of DILD in patients with lung cancer concomitant with preexisting ILD [10, 11, 12]. HMGB1 is an endogenous ligand that facilitates the activation of proinflammatory intracellular signaling associated with tumorigenesis and acute lung injury [13, 14, 15]. Based on these data, we hypothesized that tumor‐bearing status and tumor progression facilitate the occurrence of cytotoxic chemotherapy‐induced lung injury by elevating HMGB1 expression.

To test our hypothesis, this study aimed to evaluate the inflammatory response in the lungs in cancer‐bearing status. In addition, the influence of cancer‐related lung inflammation on DILD was investigated using bleomycin (BLM). Finally, the association between HMGB1 and the inflammatory response in the lungs in the tumor‐bearing status was evaluated.

## 2 Materials and Methods

### 2.1 Mice

Male C57BL/6 and B6D2F1/Crl mice (8–11 weeks old) were purchased from the Jackson Laboratory Japan (Yokohama, Japan). The animals were housed under standard conditions (five mice/cage) at our animal facility, and the experimental procedures were approved by the Committee on Animal Research at Hiroshima University (approval no. A22‐198 and A25‐46). The animals were raised under pathogen‐free conditions with a 12‐h light–dark cycle and free access to food and water. Prior to use, the animals were acclimatized to the animal care unit for at least 6 days. Before the study, the mice were anesthetized with an intraperitoneal injection of midazolam (4 mg/kg; Sandoz K.K., Tokyo, Japan), butorphanol (5 mg/kg; Meiji Seika Pharma, Yokohama, Japan), and medetomidine 0.3 mg/kg (Kyoritsu Seiyaku Corp., Tokyo, Japan) [16, 17, 18, 19]. Mice were monitored for health and behavior every other day for the duration of the experiment. To obtain lung tissues and blood sample at the end of experiments, animals were sacrificed under anesthesia of midazolam, medetomidine, and butorphanol with exsanguination/cardiac perfusion. Humane endpoint criteria were set for all animals such that any animals exhibit persistent lying down and crouching were euthanized immediately by inhalation of isoflurane (Fujifilm Wako Junyaku, Osaka, Japan). Death was confirmed based on the absence of pain responses, complete cessation of cardiac and respiratory activity and lack of corneal reflex.

### 2.2 Cells and Cell Culture

Lewis lung carcinoma (LLC) cells (American Type Culture Collection, Manassas, VA, USA), KLN205 murine squamous carcinoma cells (Cell Resource Center for Biomedical Research Institute of Development, Aging, and Cancer, Tohoku University, Miyagi, Japan), and the mouse microvascular endothelial cell line (MS1; MILE SVEN 1, ATCC CRL‐2279) were cultured in Dulbecco's modified Eagle medium (Gibco, Grand Island, NY, USA) supplemented with 10% fetal bovine serum (Sigma‐Aldrich, St. Louis, MO, USA) and 1% penicillin/streptomycin (Gibco, Grand Island, NY, USA). Cells were maintained at 37°C in a humidified atmosphere containing 5% CO_2_.

### 2.3 BLM Treatment of Tumor‐Bearing Mouse Model

Mice with matching weights were randomized, and 25 μL Hanks' Balanced Salt Solution (Gibco, Grand Island, NY, USA) and 25 μL growth factor‐reduced Matrigel matrix (Corning Life Sciences, NY, USA) with or without tumor cells (LLC 1.0 × 10^6^ cells and KLN205 3.0 × 10^6^ cells) were subcutaneously injected into the left back of each mouse under anesthesia. The appearance and growth of the tumors were monitored. The tumor volume (mm^3^) was calculated using the following formula: tumor volume = (length × width^2^)/2.

Mice were anesthetized, and BLM (Nippon Kayaku Co., Tokyo, Japan) was administered to mice using osmotic minipumps (model 2001, DurECT Corp., Cupertino, USA) containing either 200 μL of saline as a vehicle or BLM for 7 days. Osmotic minipumps were inserted in the right midback subcutaneous lesion at selected time points. Osmotic minipumps were designed to deliver their contents at 0.5 μL/h for 7 days, which were removed on days 9–11 after the BLM instillation under anesthesia. Individual body weights of mice were recorded by using an electronic balance. Four to six mice in each group were sacrificed 14 days after pump implantation.

### 2.4 Model of HMGB1‐Induced Lung Inflammation

Male C57BL/6 mice were randomly assigned to one of two groups: control or HMGB1 groups. The control models were intraperitoneally administered with 100 μL of phosphate‐buffered saline (PBS) (Nacalai Tesque, Kyoto, Japan) 5 days a week. The HMGB1 protein was sponsored by Shino‐Test Corporation (Tokyo, Japan). The HMGB1 protein was administered to the HMGB1 group by a single intraperitoneal injection of 100 μL PBS containing 100 μg/mL HMGB1 protein. Five mice in each group were sacrificed at 21 days after initiation of HMGB1 administration.

### 2.5 Effect of HMGB1 Protein on Endothelial Cells

MS1 cells were incubated with HMGB1 (1000 ng/mL) for the indicated times according to the previous studies [20, 21]. Cells were harvested using the TRIzol Reagent (Invitrogen, Carlsbad, CA, USA) and subjected to reverse transcription‐quantitative polymerase chain reaction (RT‐qPCR).

### 2.6 Bronchioalveolar Lavage Procedure

At selected time points, bronchioalveolar lavage (BAL) was performed once on each mouse. Bronchioalveolar lavage fluid (BALF) was obtained by introducing 0.5 mL of PBS into the lung and promptly drawing it out again, and these procedures were repeated three times. BALF was centrifuged at 300 × *g* for 5 min at 4°C. The total BALF cells were suspended in 1000 μL DMEM and counted using a TC20 automated cell counter (cat. no. 145‐0101J1; Bio‐Rad Laboratories, Hercules, CA, USA). The cells were cytospun onto glass slides using Cytospin (Thermo Fisher Scientific, Waltham, MA, USA), and stained with Diff‐Quick (Kokusai Shiyaku, Kobe, Japan) for cell classification. At least 300 cells per mouse were examined to quantify cell differentiation of BALF using a microscope with a ×40 objective.

### 2.7 Histology

The lungs and tumors of the mice were harvested at selected time points. Each lung and tumor was fixed in a 2% formalin solution (Natalia Tesque, Kyoto, Japan) (48 h; 4°C) and embedded in paraffin and cut into 5‐μm sections. Subsequently, the tumor sections were stained with anti‐HMGB1 antibody (1:400 dilution, cat#6893; Cell Signaling Technology, Danvers, MA, USA). Stained slides were scanned using a microscope (cat. no. BZ‐9000; Keyence, Osaka, Japan) at ×40 magnification. The lung sections were stained with hematoxylin (Muto‐Kagaku, Tokyo, Japan) and eosin (Muto‐Kagaku, Tokyo, Japan). The alveolar wall thickness was evaluated as described previously [22]. Three randomly selected regions were imaged and captured at a magnification of ×40 and incorporated a 10 μm divisions calibration slide (cat. no. AX0001; Olympus Co., Tokyo, Japan) as size bars. Images were quantified using ImageJ software (National Institutes of Health, Bethesda, MD, USA). All the images were calibrated to the same scale. Eight measurements of alveolar wall thickness were recorded for each image.

### 2.8 Flow Cytometry

Sample collection and flow cytometry analyses were performed as previously described [23]. The entire right lungs were removed, minced with sterile scissors, and incubated with collagenase A (1 mg/mL; Roche, Basel, Switzerland) for 30 min at 37°C in Roswell Park Memorial Institute 1640 medium (Gibco, Grand Island, NY, USA). Red blood cells were lysed using an ammonium‐chloride‐potassium lysis buffer (Thermo Fisher Scientific, Waltham, MA, USA). The cells were then incubated with anti‐mouse CD16/32 antibody (FcγR, clone 93; cat. no. 101302; BioLegend, San Diego, CA, USA) to block non‐specific bindings, followed by the addition of the following antibodies: from BioLegend (San Diego, CA, USA); anti‐mouse CD31 (1:8 dilution, Clone MEC13.3; cat. no. 102509), anti‐mouse CD45 (1:20 dilution, Clone 30‐F11; cat. no. 103125). Antibody‐labeled cells were washed before analysis using a flow cytometer. Flow cytometry was performed on a BD FACS Aria II (BD Biosciences, USA) or BD LSR Fortessa X‐20 system (BD Biosciences, USA). Flow cytometry data were analyzed using the FlowJo software (Tree Star Inc., Ashland, OR, USA).

### 2.9 RT‐qPCR

Total RNA was extracted using the RNeasy Mini Kit (cat. no. 74106; Qiagen, Hilden, Germany) according to the manufacturer's instructions. The quality of total RNA samples was confirmed using a spectrophotometer (NanoDrop One/One^c^; cat. no. ND‐ONE‐W; Thermo Fisher Scientific, Waltham, MA, USA). Complementary DNA was generated from the extracted RNA using a High‐Capacity RNA‐to‐cDNA Kit (cat. no. 4387406; Applied Biosystems, Foster City, CA, USA). The relative expression levels of PAI‐1, vWF, and ICAM‐1, which are associated with coagulation and leukocyte adhesion [24, 25, 26], were evaluated using the CFX96 Touch Real‐Time PCR Detection System (cat. no. 1855196J1; Bio‐Rad Laboratories, Hercules, CA, USA) and TaqMan Gene Expression Master Mix (cat. no. 4364103; Thermo Fisher Scientific, Waltham, MA, USA). PCR was performed as follows: 30 cycles at 94°C for 120 s, 98°C for 10 s, 60°C for 30 s, and 68°C for 120 s. The following primers from Applied Biosystems were used: ICAM‐1, TaqMan Gene Expression Assay ID Mm00516023_m1; PAI‐1, Mm00435858_m1; vWF, Mm00550376_m1; beta‐actin, Mm02619580_g1.

### 2.10 Measurement of HMGB1 in Serum and Culture Supernatants

HMGB1 concentrations in serum samples from mice and cell culture supernatants were evaluated using an ELISA kit (cat. no. 326054329; Shino‐Test Corporation, Tokyo, Japan). Serum samples were obtained from clotted blood after centrifugation at 1200 *g* for 20 min. LLC and KLN 205 cells were seeded at 0.1 × 10^5^ or 0.5 × 10^5^ cells per well in 24‐well plates, and the cell culture supernatants were collected after 24 h. The serum and cell culture supernatants were stored at −20°C until analysis.

### 2.11 Statistical Analysis

Data were expressed as the means ± standard errors of the means (SEM) or medians with interquartile ranges (IQR). Mann–Whitney *U*‐test was performed for comparisons between two groups, and multiple Mann–Whitney *U*‐test with Bonferroni correction were performed for comparisons between multiple groups. Spearman's correlation coefficient was used to investigate the correlation. *p*‐values < 0.05 indicated statistical significance. All values were analyzed using the JMP Pro 18.1 software (SAS Institute Inc., Cary, NC, USA).

## 3 Results

### 3.1 Evaluation of Lung Proinflammatory State in Subcutaneous Tumor‐Bearing Mice

To investigate the influence of extrapulmonary malignant tumors on the lung parenchyma, lungs from subcutaneous LLC and KLN205 tumor models in C57BL/6J and B6D2F1/Crl mice were obtained on day 21 and day 56, respectively. Serial increases in subcutaneous tumor volume in the models are presented in Figure S1. The LLC model showed more rapid tumor growth than the KLN205 model.

**FIGURE 1:**
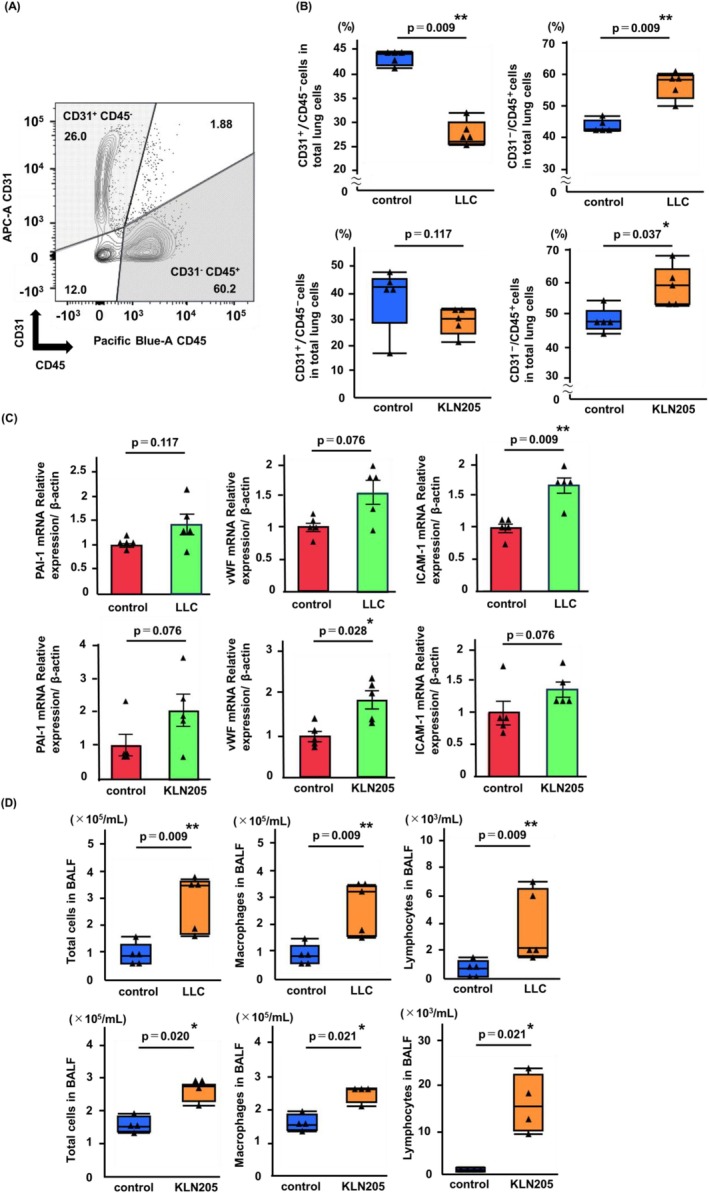
Endothelial activation and inflammatory cell infiltration in the lung of tumor‐bearing mice. (A) Expression of CD31 and CD45 in lung cells was analyzed using flow cytometry. Endothelial cells and leukocytes were defined as cells expressing CD31^+^/CD45 and CD31^−^/CD45^+^, respectively. (B) Comparison of the percentages of endothelial cells (CD31^+^/CD45^−^) and leukocytes (CD31^−^/CD45^+^) in the lungs between LLC model and KLN205 model, and control mice. (C) Expression of markers associated with endothelial activation (PAI‐1, vWF, and ICAM‐1) in pulmonary endothelial cells isolated from LLC model and KLN205 model. (D) Total cells, macrophages, and lymphocytes were significantly elevated in BALF from LLC model and KLN205 model than in controls. Data are expressed as the means ± standard errors of the means (C) or median with IQR (B, D), and analyzed for statistical significance using the Mann–Whitney *U*‐test (*n* = 4–5 mice/group). **p < 0.01, *p < 0.05. BALF, bronchoalveolar lavage fluid; IQR, interquartile range; LLC, Lewis lung carcinoma; SEM, standard errors of the means.

The CD31^+^/CD45^−^ and CD31^−^/CD45^+^ cells were defined as endothelial cells and leukocytes, respectively (Figure 1A). The gating strategies are shown in Figure S2. Flow cytometry analysis revealed that, compared with control mice, the percentage of endothelial cells (CD31^+^/CD45^−^) was significantly lower (*p* = 0.009), and the percentage of leukocytes (CD31^−^/CD45 ^+^) was significantly higher (*p* = 0.009) in the lungs of LLC model mice (Figure 1B). In the lungs of KLN205 model, the percentage of leukocytes (CD31^−^/CD45^+^) was significantly higher than that in control mice (*p* = 0.037), with no significant decrease in the percentage of endothelial cells (CD31^+^/CD45^−^) (Figure 1B).

Subsequently, to explore the cause of the significant increase in leukocytes in the tumor‐bearing murine lungs, the mRNA expression associated with endothelial activation was evaluated. RT‐qPCR results revealed a significant elevation of ICAM‐1 expression (*p* = 0.009; Figure 1C) and a trend toward increased vWF expression in endothelial cells from the lungs of the LLC model. No significant increase was observed in PAI‐1 expression. In the endothelial cells obtained from the lungs of the KLN205 model, a significant upregulation of vWF (*p* = 0.028; Figure 1C) and a trend toward increased PAI‐1 and ICAM‐1 expression were observed.

To examine inflammatory cell infiltration in the alveolar space of tumor‐bearing mice, BAL was performed. The total number of cells, macrophages, and lymphocytes in the BALF of the LLC model was significantly increased (*p* = 0.009, *p* = 0.009, and *p* = 0.009, respectively; Figure 1D). Similarly, the total number of cells, macrophages, and lymphocytes in the BALF of the KLN205 model was significantly increased (*p* = 0.021, *p* = 0.021, and *p* = 0.021, respectively; Figure 1D).

### 3.2 BLM Dosage to Mimic Treatment for Patients With Cancer

BLM was used in our experiments to imitate systemic chemotherapy in patients with cancer. In clinical practice, the dose of systemic chemotherapy is set with consideration of safety; if dose limited toxicity (DLT) including drug‐induced lung injury is observed, the dose is reduced to the amount that does not cause DLT. However, even the dose of systemic chemotherapy used in daily clinical practice induces asymptomatic inflammatory cell infiltration in BALF obtained from patients with cancer [27].

**FIGURE 2:**
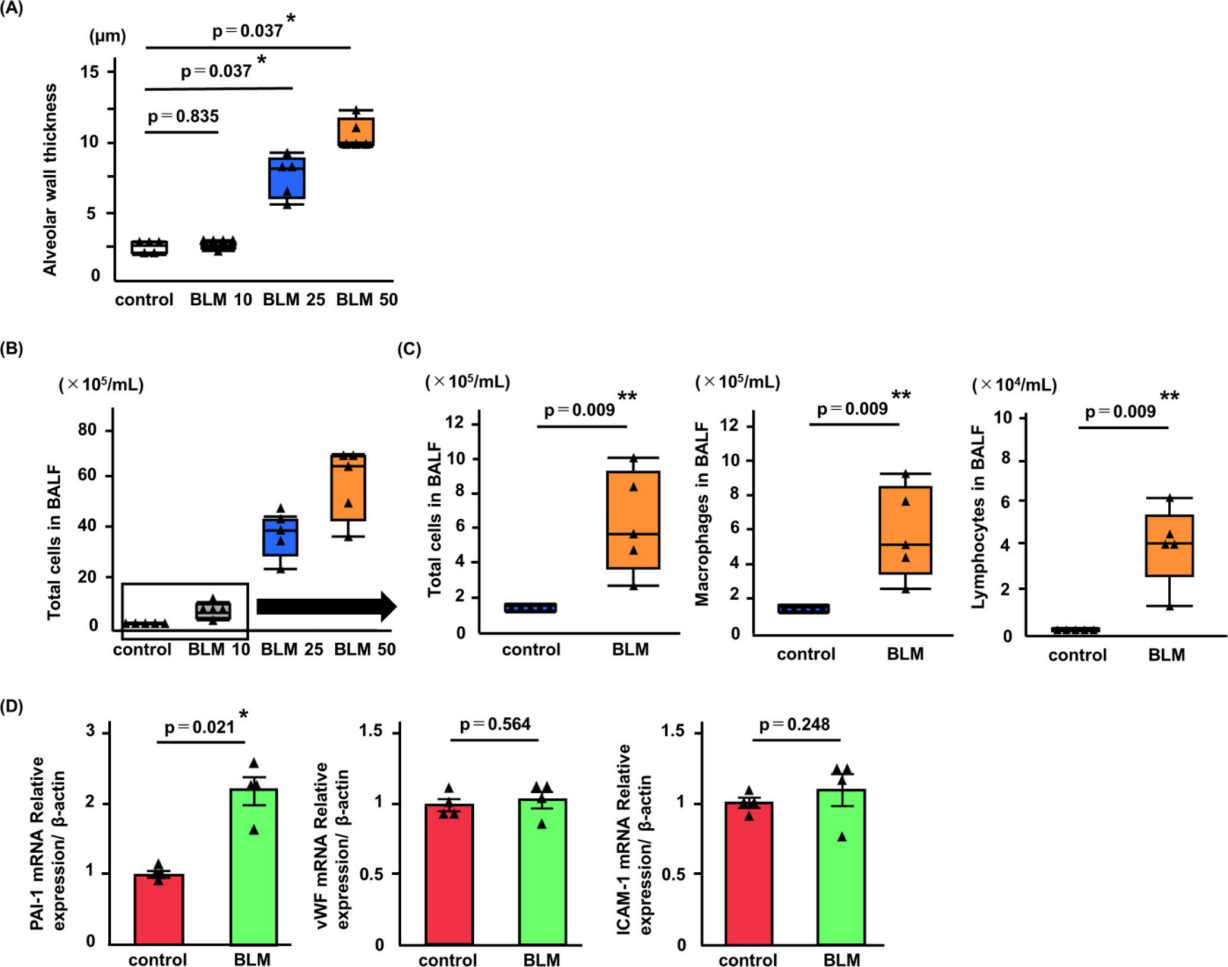
Identification of BLM dosage to mimic treatment for patients with cancer. (A) Histological analysis demonstrated substantial differences between the control and high‐dose BLM (50 mg/kg and 25 mg/kg) groups (BLM 50 and BLM 25), but not between the control and BLM 10 mg/kg (BLM 10) groups. (B) Total cell counts in BALF were lower in mice treated with BLM 10 mg/kg (BLM 10) than in mice treated with high‐dose BLM groups (BLM50 or BLM25). (C) However, total cell counts, macrophages, and lymphocytes in BALF from BLM 10 group were significantly higher than in the saline‐treated mice (control). (D) Expression of markers associated with endothelial activation (PAI‐1, vWF, and ICAM‐1) in pulmonary endothelial cells isolated from BLM group. Only PAI‐1 showed significant increases in gene expression. Data are expressed as median with IQR (A–C) or means ± SEM (D) and analyzed for statistical significance using the Mann–Whitney *U*‐test or multiple Mann–Whitney *U*‐test with Bonferroni correction (*n* = 4–5 mice/group). **p < 0.01, *p < 0.05. BALF, bronchoalveolar lavage fluid; BLM, bleomycin; IQR, interquartile range; SEM, standard errors of the means.

To identify the BLM dose that mimicked the clinical situation, BLM was administered to the mice at doses of 10, 25, and 50 mg/kg. BLM 50 mg/kg and 25 mg/kg groups induced body weight loss, while the trend in body weight in the BLM 10 mg/kg group was comparable to the control group (Figure S3). Histological alveolar wall thickening was observed in the BLM 50 and BLM 25 groups (Figure 2A). On the other hand, no significant histological differences were observed between the control group and BLM 10 group (Figure 2A). In flow cytometry analysis, no significant difference was observed in the percentage of endothelial cells (CD31^+^/CD45^−^) and leukocytes (CD31^−^/CD45 ^+^) in the lungs of BLM‐treated mice compared to control mice (Figure S4). However, although inflammatory cell infiltration in the BLM 10 group was lower than that in the BLM 50 or 25 groups (Figure 2B), the numbers of total cells, macrophages, and lymphocytes in the BALF of the BLM 10 group were significantly increased compared to the control group (*p* = 0.009, *p* = 0.009, and *p* = 0.009, respectively; Figure 2C). Therefore, based on these results, we selected a dose of 10 mg/kg to imitate cancer treatment.

In addition, the proinflammatory activity in pulmonary endothelial cells was analyzed in BLM 10 group. Pulmonary endothelial cells isolated from BLM‐treated mice on day 21 were analyzed using RT‐qPCR, which revealed significant differences in the gene expression levels of PAI‐1 compared to controls (*p* = 0.021). However, no significant changes in the expression levels of vWF and ICAM‐1 were observed (Figure 2D). Additionally. Serum levels of HMGB1 were not significantly elevated in BLM 10 group compared to controls (data not shown).

### 3.3 Tumor‐Bearing Status Aggravates Endothelial Proinflammatory Change and Inflammatory Cell Infiltration in the Lungs Induced by BLM

**FIGURE 3:**
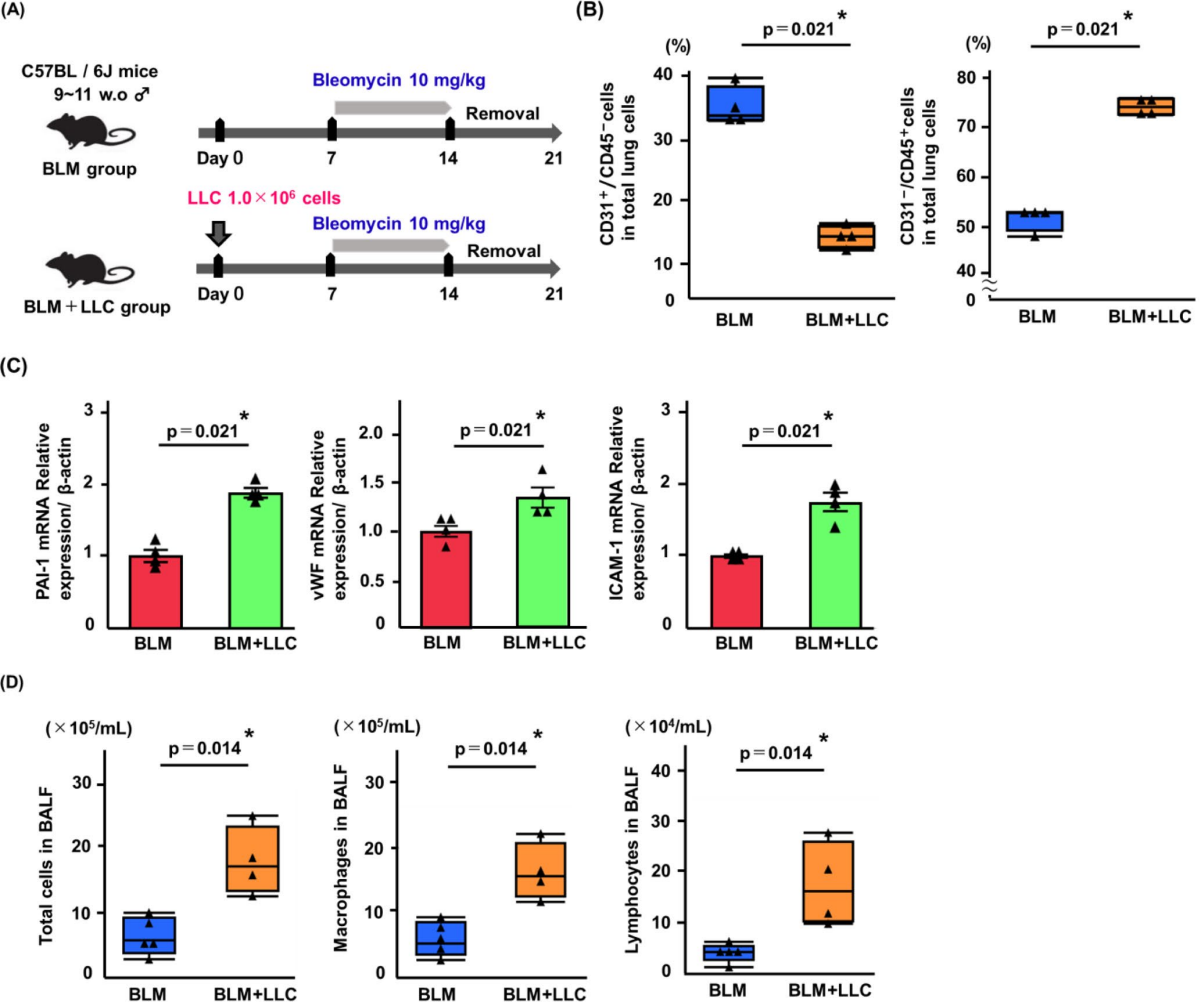
Tumor‐bearing status aggravates endothelial proinflammatory change and inflammatory cell infiltration in lungs induced by BLM. (A) Experimental scheme. Male mice were used. BLM group mice were implanted with osmotic minipumps containing BLM 10 mg/kg, and BLM + LLC group mice were subcutaneously implanted with 1.0 × 10^6^ LLC cells and pumps containing BLM 10 mg/kg. (B) Compared with the BLM group, endothelial cells (CD31^+^/CD45^−^) were significantly decreased, and leukocytes (CD31^−^/CD45^+^) were increased in BLM + LLC group. (C) Compared with the BLM group, pulmonary endothelial cells isolated from BLM + LLC group exhibited significantly elevated expression of markers associated with endothelial activation (PAI‐1, vWF, and ICAM‐1). (D) Total cells, macrophages, and lymphocytes were significantly increased in BALF from BLM + LLC group, compared with those in BLM group. Data are expressed as median with IQR (B, D) or means ± SEM (C) and analyzed for statistical significance by Mann–Whitney *U*‐test (*n* = 4–5 mice/group). ***p < 0.001, **p < 0.01, *p < 0.05. BLM, bleomycin; BALF, bronchoalveolar lavage fluid; IQR, interquartile range; SEM, standard errors of the means.

Pulmonary endothelial activation in tumor‐bearing mice treated with BLM was assessed. The experimental scheme is illustrated in Figure 3A. The percentage of pulmonary endothelial cells (CD31^+^/CD45^−^) was significantly lower in the BLM + LLC group than that observed in mice treated with BLM alone (BLM group) (*p* = 0.021; Figure 3B). The percentage of leukocytes (CD31^−^/CD45^+^) in the BLM + LLC group was significantly higher than that in the BLM group (*p* = 0.021; Figure 3B). In the pulmonary endothelial cells from the BLM + LLC group, the relative expression levels of PAI‐1, vWF, and ICAM‐1 were significantly higher than those in the BLM group (PAI‐1, *p* = 0.021; vWF, *p* = 0.021; ICAM‐1, *p* = 0.021; Figure 3C). BALF analysis revealed significant increases in the numbers of total cells, macrophages, and lymphocytes in the BLM + LLC group than in the BLM group (*p* = 0.014, *p* = 0.014, and *p* = 0.014, respectively; Figure 3D).

**FIGURE 4:**
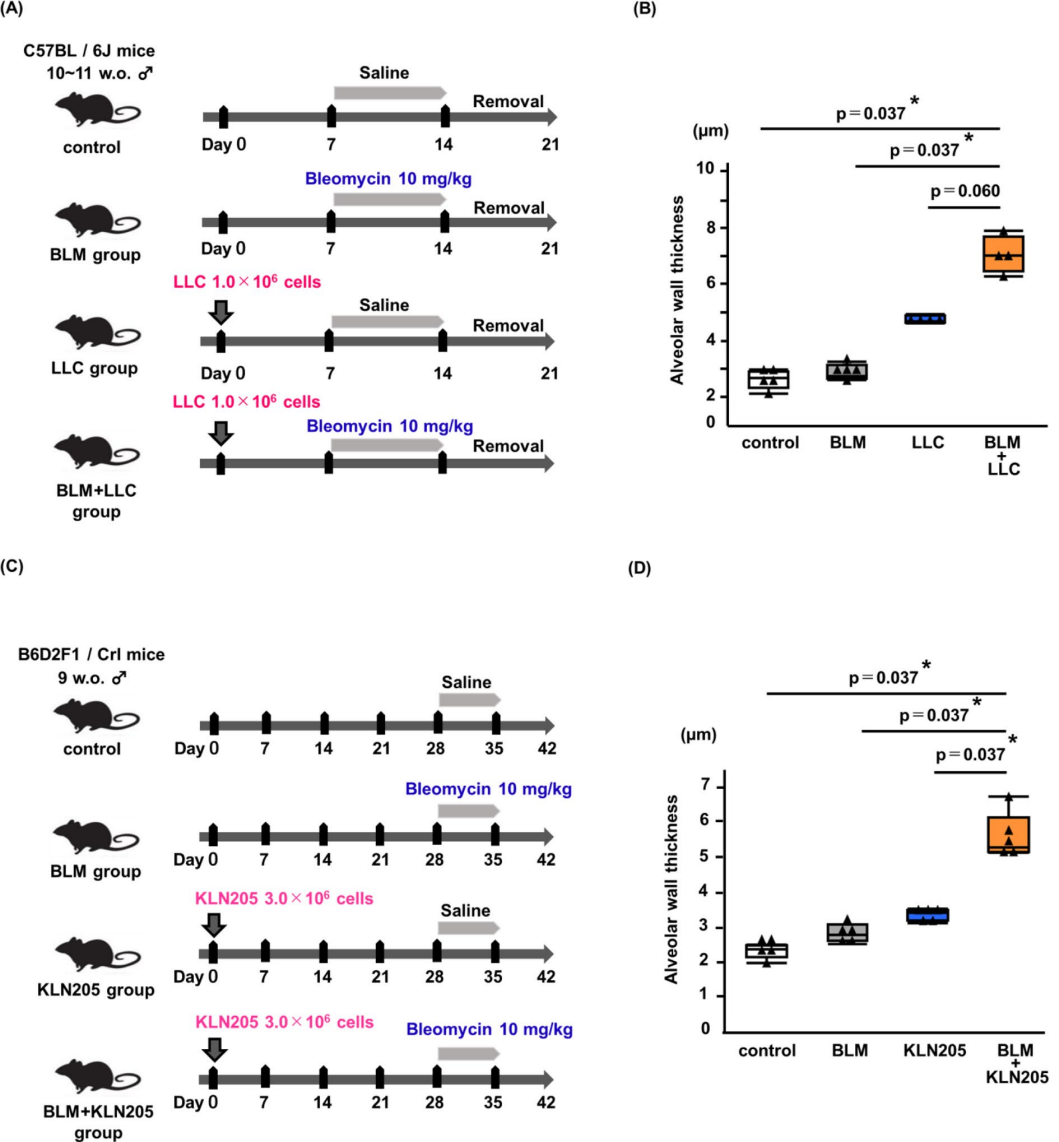
Evaluation of alveolar wall thickness in BLM‐induced tumor‐bearing status. (A) Experimental scheme. Based on the proposed treatment, mice were divided into the following four groups: Control group (mice implanted with osmotic minipumps containing saline), BLM group (mice implanted with osmotic minipumps containing BLM 10 mg/kg) LLC group (mice subcutaneously implanted with 1.0 × 10^6^ LLC cells and implanted with osmotic minipumps containing saline), and BLM + LLC group (mice subcutaneously implanted with 1.0 × 10^6^ LLC cells and implanted with osmotic minipumps containing BLM 10 mg/kg). (B) Semi‐quantitative analysis demonstrated that, compared with other groups, BLM + LLC group exhibited increased alveolar wall thickness on day 21. (C) Experimental scheme. Based on the proposed treatment, mice were divided into the following four groups: Control group (mice implanted with osmotic minipumps containing saline), BLM group (mice implanted with osmotic minipumps containing BLM 10 mg/kg), KLN205 group (mice subcutaneously implanted with 3.0 × 10^6^ KLN205 cells and osmotic minipumps containing saline), and BLM + KLN205 group (mice subcutaneously implanted with 3.0 × 10^6^ KLN205 cells and osmotic minipumps containing BLM 10 mg/kg). (D) In KLN205 model, histological changes similar to the LLC model were observed. Data are expressed as median with IQR (B, D) and analyzed by multiple Mann–Whitney *U*‐test with Bonferroni correction (*n* = 4–5 mice/group). ***p < 0.001, **p < 0.01, *p < 0.05. ANOVA, analysis of variance; BLM, bleomycin; IQR, interquartile range.

To further characterize the pulmonary changes in tumor‐bearing mice treated with BLM, we performed experiments summarized in Figure 4A. Alveolar wall thickness was measured and those were significantly increased in the BLM + LLC group than in the control group and BLM group (*p* = 0.037, *p* = 0.037, respectively; Figure 4B). We found a trend toward increased alveolar wall thickness in the BLM + LLC group compared to the LLC group (*p* = 0.060; Figure 4B). A similar experiment was performed using the KLN205 cells (Figure 4C). Corroborating the findings from the LLC experiments, the BLM + KLN205 group exhibited increased alveolar wall thickness than the other groups (*p* = 0.037 vs. control, *p* = 0.037 vs. BLM group, *p* = 0.037 vs. KLN205 group; Figure 4D).

### 3.4 Tumor‐Associated HMGB1 Induces Inflammatory Cell Infiltration in the Lung and Endothelial Activation

**FIGURE 5:**
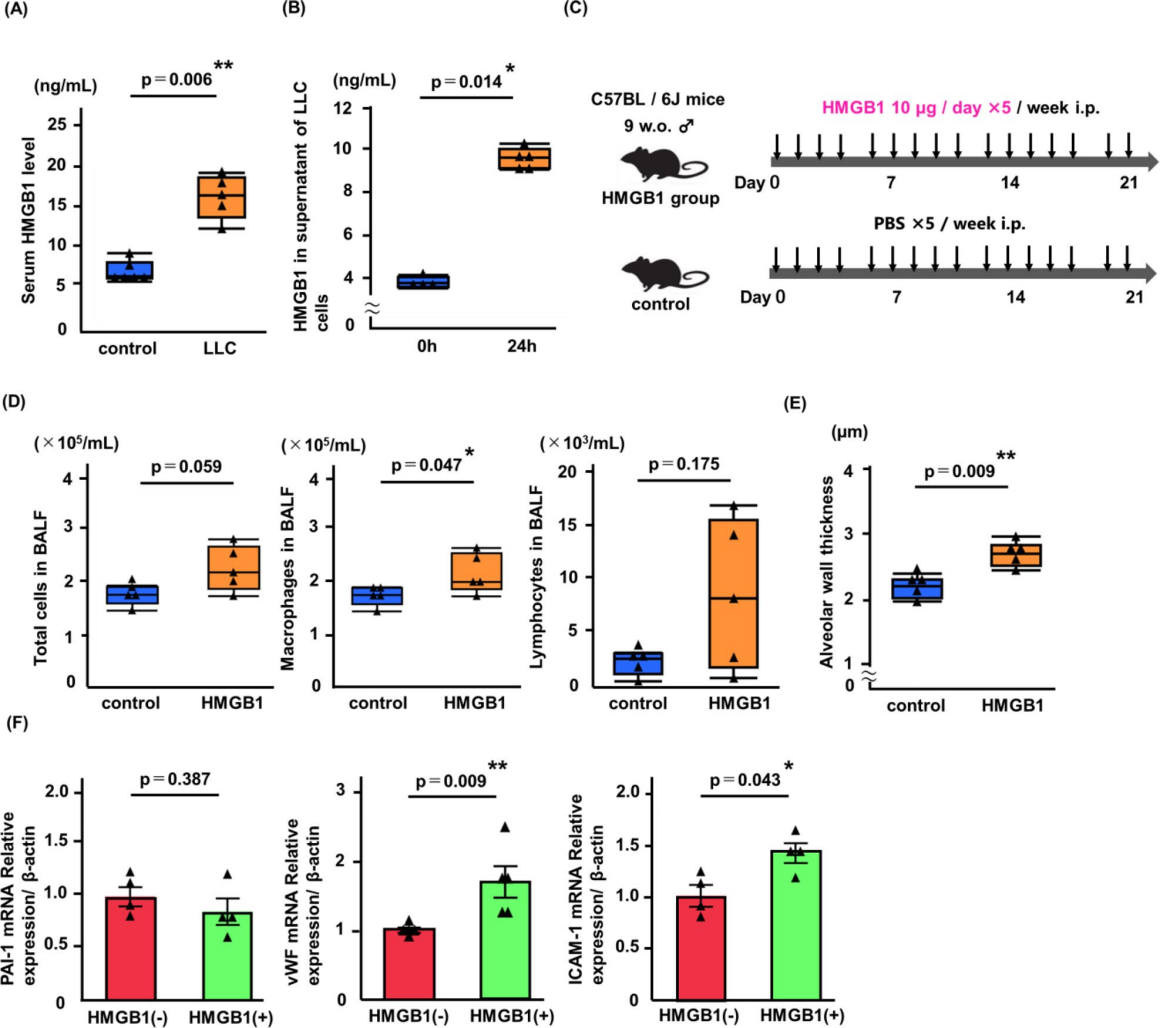
Tumor‐associated HMGB1 induces inflammatory cell infiltration in lungs and proinflammatory activation on endothelial cells. (A) Serum HMGB1 levels in LLC model were significantly higher than in control mice. (B) In vitro LLC cells secreted HMGB1 into cell culture medium (*n* = 4–5/group). (C) Experimental scheme. HMGB1 group mice were intraperitoneally administered PBS containing HMGB1 protein and control group mice were intraperitoneally administered PBS. (D, E) Total cell counts in BALF and alveolar wall thickness in lung of HMGB1 group were significantly higher compared with those in the control group (*n* = 5 mice/group). (F) mRNA levels of vWF and ICAM‐1, but not PAI‐1, were elevated in MS1 cells cultured with HMGB1 protein (1000 ng/mL) (*n* = 4–5/group). Data were expressed as median with IQR (A, B, D, and E) or means ± SEM (F), and analyzed for statistical significance by Mann–Whitney *U*‐test. **p < 0.01, *p < 0.05. BALF, bronchoalveolar lavage fluid; HMGB1, high‐mobility group box 1; i.p., intraperitoneal injection; IQR, interquartile range.

To verify the involvement of HMGB1 in the proinflammatory changes in the lungs, the levels of HGMB1 in the serum of tumor‐bearing mice were measured. Blood samples were collected from subcutaneous LLC and KLN205 tumor‐bearing models on day21 and day 42, respectively. When compared to the control group, serum HMGB1 levels in LLC model were significantly elevated (*p* = 0.006; Figure 5A), and a significant and positive correlation was observed between tumor volume and serum HMGB1 concentration (*r*_*s*_ = 0.8359, *p* = 0.007; Figure S5A). In KLN205 model, there was a significant and positive correlation between tumor volume and serum HMGB1 concentration (*r*_*s*_ = 0.8503, *p* = 0.008; Figure S5B), although there was no significant increase in serum HMGB1 levels compared to the control group (Figure S6A). To identify the source of HMGB1 in tumor‐bearing mice, HMGB1 expression in subcutaneous tumors was evaluated by immunohistochemistry (IHC) staining. Immunohistochemical results showed that HMGB1 was expressed in the nucleus and cytoplasm of tumor cells in both LLC and KLN205 models (Figure S7). Next, the change in HMGB1 concentration in the culture supernatants of these lung cancer cell lines was analyzed. Elevated HMGB1 levels were detected in the culture supernatants of LLC (*p* = 0.014; Figure 5B) and KLN205 cells (*p* = 0.030; Figure S6B).

Subsequently, the effect of HMGB1 on inflammatory cell infiltration in the lungs was evaluated. The study design is summarized in Figure 5C. Compared with control mice, a trend toward the increased numbers of total inflammatory cells and the significant increased numbers of macrophages in the BALF were observed in mice that received an intraperitoneal injection of HMGB1 protein (*p* = 0.059 and *p* = 0.047, respectively; Figure 5D). No significant change in the numbers of lymphocytes was found. Further, a significant increase in alveolar wall thickness was observed in HMGB1‐treated mice compared to control mice (*p* = 0.009; Figure 5E). To elucidate the role of HMGB1 in the regulation of endothelial proinflammatory activation, the murine endothelial cell line MS1 was cultured in a medium supplemented with HMGB1 protein. As shown in Figure 5F, incubation of MS1 cells with HMGB1 resulted in significantly increased expression of vWF and ICAM‐1 (vWF, *p* = 0.009; ICAM‐1, *p* = 0.043; Figure 5F), although the mRNA expression of PAI‐1 did not increase.

## 4 Discussion

Our findings demonstrated that tumor‐bearing status accelerated inflammatory cell infiltration into the lungs. Moreover, tumor‐bearing status additively aggravated BLM‐induced inflammatory cell infiltration and alveolar wall thickening. Inflammatory cell infiltration caused by tumor‐bearing status could be promoted by endothelial activation, in which tumor‐associated HMGB1 production might be involved.

This study demonstrated that extrapulmonary tumors significantly increased inflammatory cell infiltration into the lungs, as evaluated using BALF. The association between tumor and lung inflammatory cell infiltration remained consistent between the LLC and KLN205 tumor models. Furthermore, we demonstrated that subcutaneous tumor‐bearing status leads to endothelial activation in the lungs in vivo. Upregulation of ICAM‐1, a leukocyte adhesion molecule, accelerates the adhesion of circulating immune cells to the pulmonary endothelium and contributes to immune cell migration and perivascular infiltration [24, 25]. PAI‐1 and vWF are associated with microvascular coagulation, an important pathophysiological event in inflammation [26]. The elevated gene expression levels of PAI‐1, vWF, or ICAM‐1 in pulmonary endothelial cells from tumor‐bearing murine models underscore the role of tumor‐induced endothelial activation in promoting lung inflammation. Taken together, these observations suggest that the presence and progression of tumors may lead to proinflammatory status, representative of endothelial activation and inflammatory cell infiltration in the lung, thereby promoting lung injury.

Next, the influence of tumor‐bearing status on BLM‐induced lung injury was evaluated. BALF and flow cytometry analyses revealed that, compared with mice treated with BLM alone, BLM‐treated tumor‐bearing mice exhibited increased inflammatory cell accumulation in the lungs. Moreover, the mRNA expression levels of PAI‐1, vWF, and ICAM‐1 in pulmonary endothelial cells from BLM‐treated tumor‐bearing mice were higher than in those from mice treated with BLM alone. Moreover, alveolar wall thickness was additively aggravated by the presence of tumors and BLM treatment. These results indicate that the presence of tumors exacerbates inflammatory cell infiltration and alveolitis induced by BLM. This finding suggests the potential for targeting molecules associated with tumorigenesis to evaluate and mitigate the risk of DILD.

HMGB1 is known to play an important role in the development of inflammation, tumorigenesis, and lung injury [13, 14, 15]. Thus, this study focused on HMGB1 to explore the association between extrapulmonary tumors and lung inflammation. First, we evaluated the expression of HMGB1 in vivo and in vitro. In vivo, the IHC staining method demonstrated that HMGB1 was highly expressed in tumors of LLC and KLN205, particularly in the nucleus and cytoplasm. Additionally, serum levels of HMGB1 were positively correlated with tumor volume. In vitro, HMGB1 was elevated in the cancer cell supernatant. These findings indicate that tumor cells may be a main source of HMGB1 in our model. HMGB1 is mainly located in the nucleus and binds to chromatin [28]. HMGB1 is acetylated and then translocated from the nucleus to the cytoplasm. In addition to acetylation, various post‐translational modifications such as methylation, phosphorylation, and oxidation regulate the translocation and release of HMGB1 to the extracellular space in response to various stresses [28, 29, 30]. Therefore, tumor progression may be associated with increasing levels of HMGB1. Second, this study also evaluated the effect of exogenous HMGB1 on mice lung and the endothelium cell line MS1. In vivo, intraperitoneal administration of HMGB1 protein in mice resulted in an increased tendency for inflammatory cell infiltration into the lung and significant thickening of the alveolar wall. In vitro, HMGB1 protein induced the expression of the mediators of endothelial activation, vWF and ICAM‐1, in the MS1 cell line. These findings indicate that elevated HMGB1 in circulation may be associated with endothelial activation that would accelerate the aggravation of DILD. On the other hand, as a limitation of the study, the in vivo and in vitro model of HMGB1 administration could not fully replicate tumor‐induced inflammatory responses. For example, the upregulation of PAI‐1 in pulmonary endothelial cells was observed in only the tumor‐bearing model, but not in vitro with HMGB1 administration. These discrepancies suggest that some molecules associated with tumors other than HMGB1 may also be involved in the association between tumor progression and DILD, and therefore further studies are required to elucidate the mechanistic association between tumor and DILD.

This study has several other limitations. First, only BLM was used in this study. Recently, many kinds of drugs for cancer treatment were reported as causes of DILD, and therefore the mechanistic association between cancer and the aggravation of DILD needs to be validated in each drug for cancer treatment. Second, the possibility of sex bias could not be ruled out in this study, as all experiments were conducted using male mice. Third, further investigations are required to determine whether these murine findings are applicable to DILD in humans.

In conclusion, tumor‐bearing status suggests a potential role in the development of BLM‐induced pulmonary inflammation through endothelial activation. Modulating these proinflammatory activities in tumor‐bearing status may provide valuable insights into how to potentially overcome drug‐induced inflammatory conditions.

## Author Contributions

S.I. contributed to the methodology, data curation, investigation, validation, formal analysis, visualization, and original draft preparation. K.Y. contributed to the methodology, visualization, formal analysis, funding acquisition, project administration, and original draft preparation. K.F. contributed to the investigation, formal analysis, and review and editing. K.S. contributed to the methodology, supervision, and review and editing. H.I., Y.H., S.S., T.M., T.N., S.O., and H.H. contributed to the supervision and review and editing. N.H. contributed to the funding acquisition, supervision, and review and editing. All the authors have read and approved the submission of the final manuscript. K.Y. and N.H. confirm the authenticity of all the raw data.

## Funding

This work received grants from Japan Society for the Promotion of Science KAKENHI (grant number 18K15951 and 22K16194).

## Ethics Statement

The experimental procedures were approved by the Committee on Animal Research at Hiroshima University (approval no. A22‐198 and A25‐46).

## Consent

The authors have nothing to report.

## Conflicts of Interest

Noboru Hattori and Kakuhiro Yamaguchi received research grants from the Shino‐test Corporation. The other authors declare no conflicts of interest.

## Supporting information


**Figure S1:** Changes in tumor volume in LLC‐ and KLN205‐bearing mice.
**Figure S2:** Flow cytometry gating strategy for pulmonary endothelial cells.
**Figure S3:** Monitoring change in body weight of BLM‐treated mice.
**Figure S4:** The percentages of endothelial cells (CD31+/CD45−) and leukocytes (CD31−/CD45+) in the lungs.
**Figure S5:** Correlation between serum levels of HMGB1 and subcutaneous tumor volume.
**Figure S6:** HMGB1 concentrations in serum of KLN205‐bearing mice and KLN205 cell culture supernatant.
**Figure S7:** Immunohistochemical expression of HMGB1 in the subcutaneous tumor tissues in mice.

## Data Availability

The data generated in the present study may be requested from the corresponding author.
