# Supplementary material for: Tumor‐Bearing Status Accelerates Bleomycin‐Induced Pulmonary Inflammation via Endothelial Activation
Source: Thorac Cancer. 2026 Mar 30;17(7):e70272. doi: 10.1111/1759-7714.70272 (PMC13070733; doi:10.1111/1759-7714.70272)
Supplement: Supplementary file 1 — Figure S1: Changes in tumor volume in LLC‐ and KLN205‐bearing mice. Figure S2: Flow cytometry gating strategy for pulmonary endothelial cells. Figure S3: Monitoring change in body weight of BLM‐treated mice. Figure S4: The percentages of endothelial cells (CD31+/CD45−) and leukocytes (CD31−/CD45+) in the lungs. Figure S5: Correlation between serum levels of HMGB1 and subcutaneous tumor volume. Figure S6: HMGB1 concentrations in serum of KLN205‐bearing mice and KLN205 cell culture supernatant. Figure S7: Immunohistochemical expression of HMGB1 in the subcutaneous tumor tissues in mice. [file TCA-17-e70272-s001.pdf]

Supplemental Figure S1:

Changes in tumor volume in LLC- and KLN205-bearing mice.

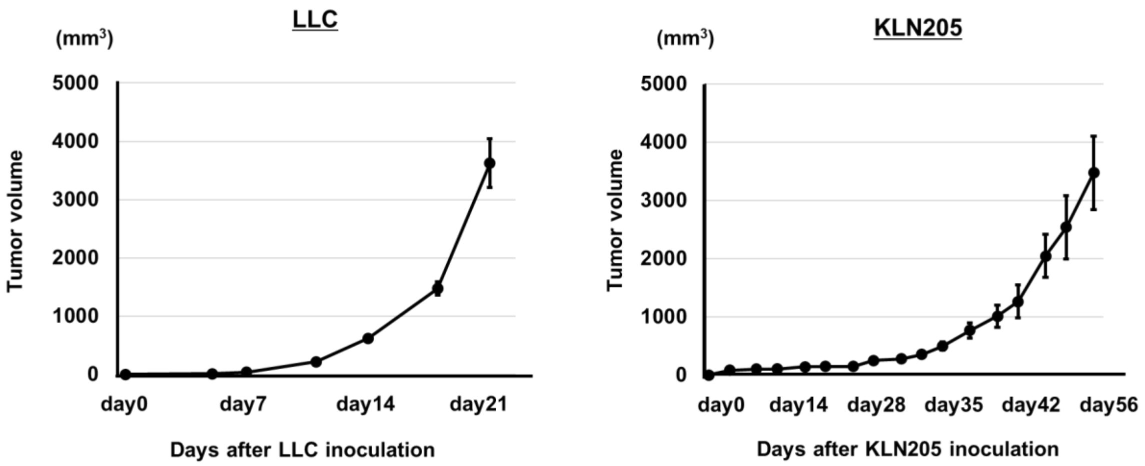

Subcutaneous tumor volume of LLC-bearing mice (left) and KLN205-bearing mice (right). Data were expressed as means ± SEM (n = 5 /group).

LLC, Lewis lung carcinoma; SEM, standard errors of the means.

Supplemental Figure S2:

Flow cytometry gating strategy for pulmonary endothelial cells.

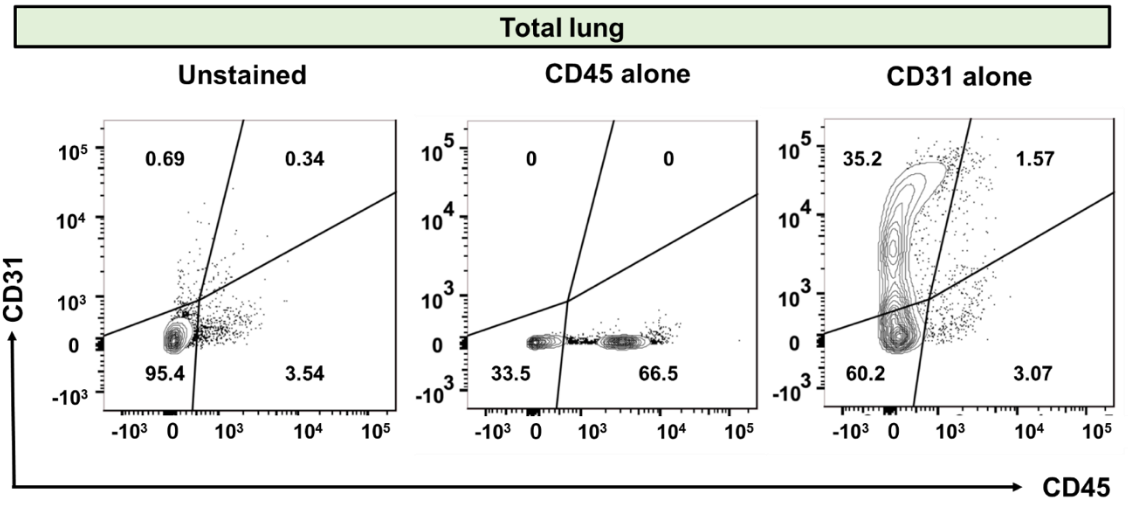

Pulmonary endothelial cells were separated from other lung cells by CD31 and CD45 gating, which was determined by comparing samples with unstained (left), with CD45 alone (middle) and CD31 alone (right).

Supplemental Figure S3:

Monitoring change in body weight of BLM-treated mice.

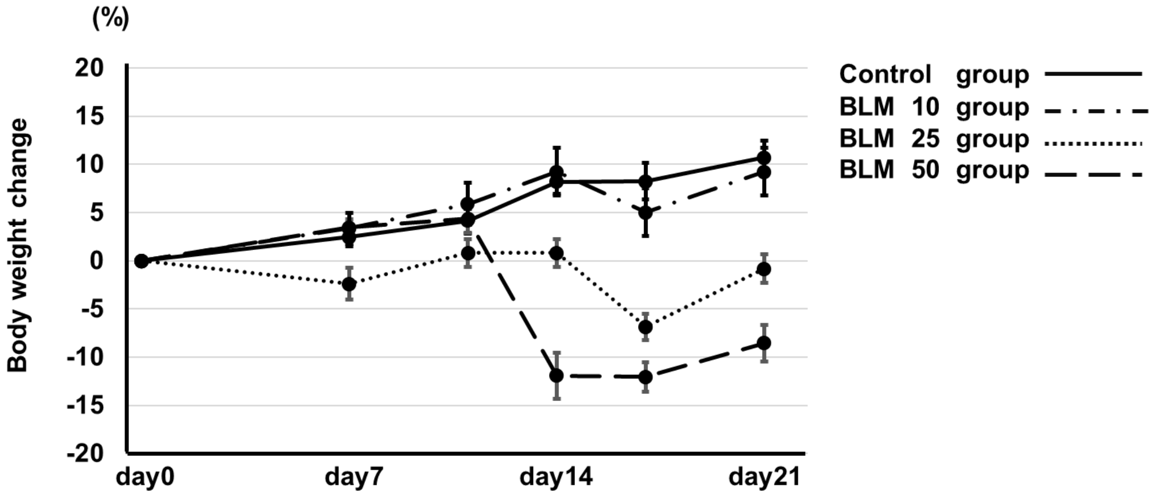

Time course of changes in body weight in mice administered BLM at 0 mg/kg(control), 10 mg/kg, 25 mg/kg and 50 mg/kg. The body weight on day 0 was defined as 0%. The relative body weight was calculated as a percentage of the value measured on day 0. Data were expressed as means ± SEM (n = 5 /group).

BLM, bleomycin; SEM, standard errors of the means.

Supplemental Figure S4:

The percentages of endothelial cells ($CD31^+/CD45^-$) and leukocytes ($CD31^-/CD45^+$) in the lungs.

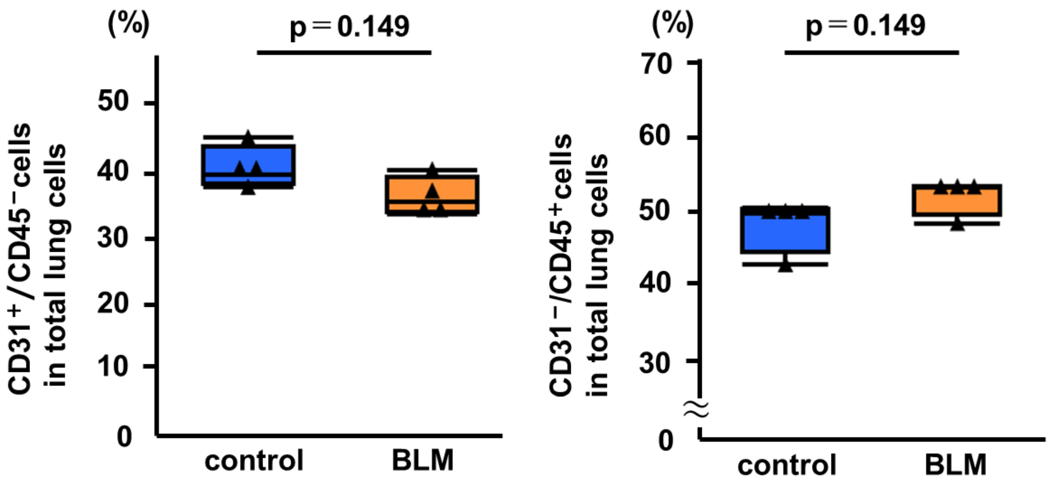

The comparison of the percentages of endothelial cells ($CD31^+/CD45^-$) (left) and leukocytes ($CD31^-/CD45^+$) (right) in the lungs between BLM model BLM group (mice implanted with osmotic minipumps containing BLM 10 mg/kg) and control mice. Data are expressed as median with IQR and analyzed for statistical significance using the Mann-Whitney *U*-test (n = 4 /group).

***$p < 0.001$, **$p < 0.01$, * $p < 0.05$.

BLM, bleomycin; IQR, interquartile range.

Supplemental Figure S5:

Correlation between serum levels of HMGB1 and subcutaneous tumor volume.

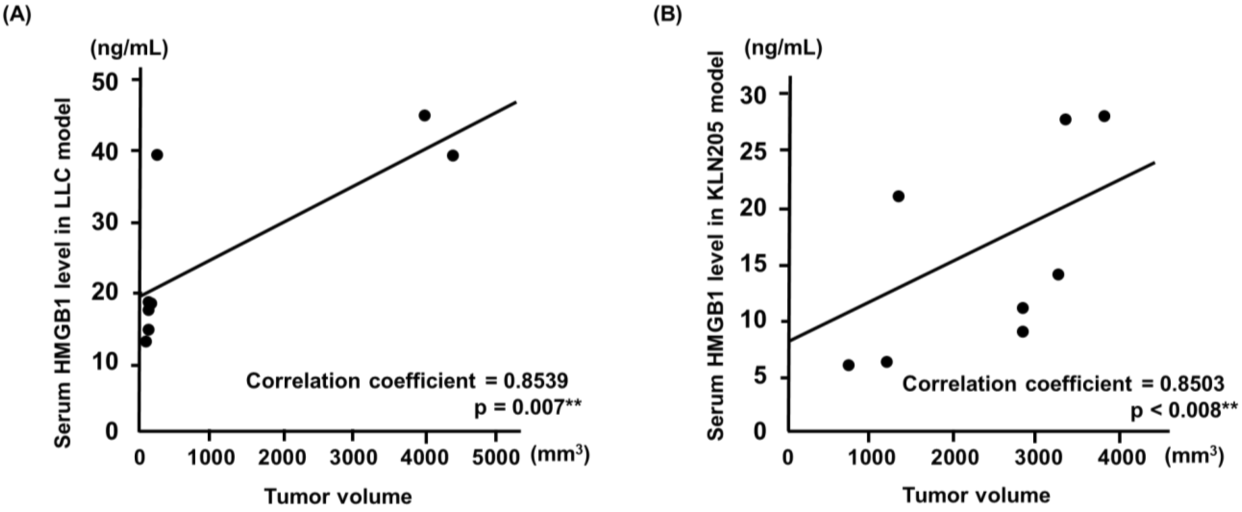

There was a significant correlation between serum HMGB1 levels and subcutaneous tumor volume in LLC (left) and KLN205 model (right) (n = 8 mice/group). Data were analyzed for statistical significance by Spearman's correlation coefficient analysis.

***p < 0.001, **p < 0.01, * p < 0.05.

HMGB1, high-mobility group box 1; LLC, Lewis lung carcinoma.

Supplemental Figure S6:

HMGB1 concentrations in serum of KLN205-bearing mice and KLN205 cell culture supernatant.

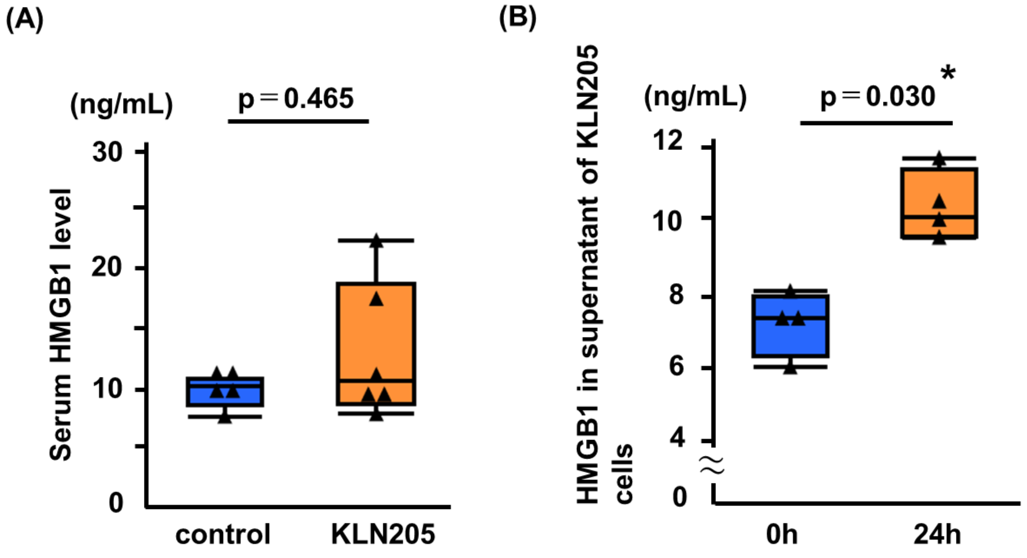

(A) Comparison of serum HMGB1 levels between KLN205 model and control mice (n = 5-6 / group). **(B)** KLN205 cells secreted HMGB1 into cell culture medium (n = 4 /group). ***$p < 0.001$, **$p < 0.01$, * $p < 0.05$.

HMGB1, high-mobility group box 1.

Supplemental Figure S7:

Immunohistochemical expression of HMGB1 in the subcutaneous tumor tissues in mice.

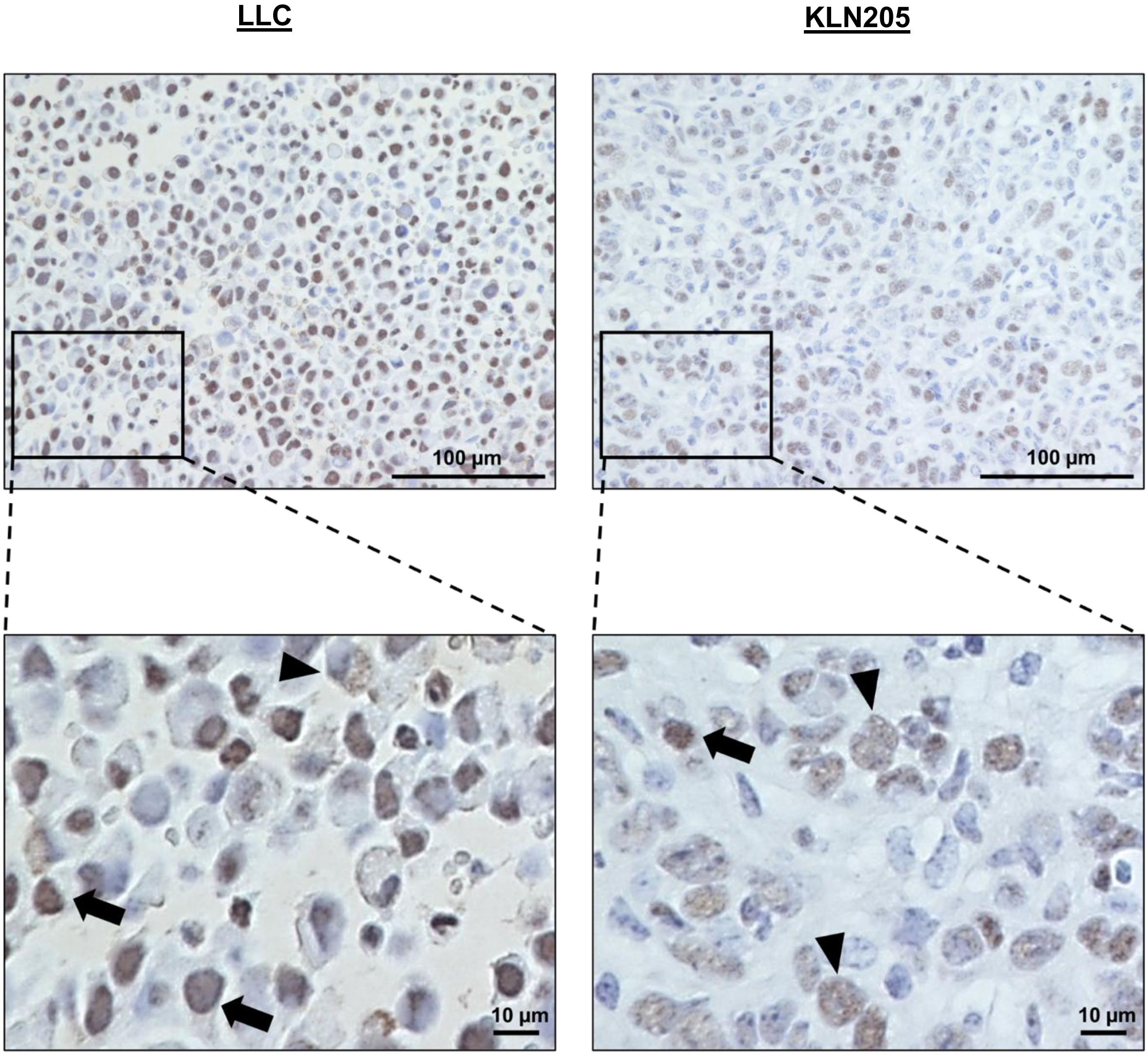

Representative immunohistochemistry images of staining for HMGB1 in the tumor tissues of LLC model (left) and KLN205 model (right). Positive expression of HMGB1 was observed in the nucleus (arrow) and cytoplasm (arrow head) in tumor cells of both models.

HMGB1, high-mobility group box 1; LLC, Lewis lung carcinoma.
